# Risque cardiovasculaire absolu des femmes sous contraception hormonale à Porto–Novo

**DOI:** 10.5830/CVJA-2018-016

**Published:** 2018

**Authors:** Sonou Arnaud, Ogoudjobi Mathieu, Houehanou Corine, Aniglé Richard, Amoussou-Guénou Daniel, Mahouna Adjagba Philippe, Hounkponou Murielle, Bognon Rosaire, Assani Salimatou, Martin Houénassi Dèdonougbo, Codjo Léopold

**Affiliations:** Centre Hospitalier Universitaire Départemental de l’Ouémé–Plateau, Porto–Novo, Bénin; Centre Hospitalier Universitaire Départemental de l’Ouémé–Plateau, Porto–Novo, Bénin; Centre Hospitalier Universitaire Départemental de l’Ouémé–Plateau, Porto–Novo, Bénin; Centre Hospitalier Universitaire Départemental de l’Ouémé–Plateau, Porto–Novo, Bénin; Centre Hospitalier Universitaire Départemental de l’Ouémé–Plateau, Porto–Novo, Bénin; Centre National Hospitalier Universitaire Hubert Koutoukou Maga, Cotonou, Bénin; Centre National Hospitalier Universitaire Hubert Koutoukou Maga, Cotonou, Bénin; Centre National Hospitalier Universitaire Hubert Koutoukou Maga, Cotonou, Bénin; Centre National Hospitalier Universitaire Hubert Koutoukou Maga, Cotonou, Bénin; Centre National Hospitalier Universitaire Hubert Koutoukou Maga, Cotonou, Bénin; Centre Hospitalier Universitaire Départemental du Borgou– Alibori, Parakou, Bénin

**Keywords:** risque cardiovasculaire absolu, contraception hormonale, eligibilite, absolute cardiovascular risk, hormonal contraception, eligibility

## Abstract

**Introduction:**

Le but du present travail etait de determiner le risque cardiovasculaire absolu (RCVA) des femmes utilisant une contraception hormonale a Porto–Novo.

**Méthode:**

Nous avons mene une etude transversale descriptive incluant des femmes au moment du renouvellement d’une methode contraceptive hormonale. La pression arterielle, la glycemie veineuse a jeun, l’indice de masse corporelle et l’hypertrophie ventriculaire gauche electrocardiographique ont ete etudiees. La determination du RCVA a ete double basee sur les modeles de l’Organisation Mondiale de la Sante (OMS/ISH) et de la Societe Europeenne de Cardiologie (ESC/ ESH).

**Résultats:**

L’age moyen des femmes etait de 35.3 ± 8.2 ans. La pression arterielle et la glycemie etaient elevees dans respectivement 24 et 1.5% des cas; 23.2% etaient obeses. L’hypertrophie du ventricule gauche etait presente dans 7.1% des cas. Un RCVA eleve etait retrouve chez 5.2% de ces femmes selon le modele de l’ESC/ESH.

**Conclusion:**

L’existence de femmes a RCVA eleve dans ce groupe souleve le probleme de l’eligibilite cardiovasculaire a la methode contraceptive utilisee.

## Abstract (en)

**Introduction:**

The purpose of this work was to determine the absolute cardiovascular risk (ACVR) of women using hormonal contraception in Porto–Novo.

**Methods:**

We carried out a descriptive, cross–sectional study, including women at the time of renewal of a hormonal contraceptive method. Blood pressure, fasting venous blood glucose level, body mass index and electrocardiographic left ventricular hypertrophy were studied. The determination of ACVR was dual based on the World Health Organisation (WHO/ISH) and the European Society of Cardiology (ESC/ ESH) models.

**Results:**

The mean age of the women was 35.3 ± 8.2 years. Blood pressure and blood glucose levels were high in 24 and 1.5% of cases, respectively. Left ventricular hypertrophy was present in 7.1% of cases. A high ACVR was found in 5.2% of these women, using the ESC/ESH model.

**Conclusion:**

The occurrence of women with high ACVR in this group raises the problem of cardiovascular eligibility to the contraceptive method used.

Les methodes contraceptives hormonales, du fait de leurs mecanismes d’action, ne sont pas sans risque sur l’organisme particulierement sur la fonction cardiovasculaire et le metabolisme glucido–lipidique.^1^ Une augmentation mineure des chiffres de pression arterielle (5 a 7 mmHg) a ete notee sous oestroprogestatifs. L’hypertension arterielle franche apparait chez 0.6 a 2.8% de ces utilisatrices.^2^ L’identification des principaux facteurs de risque atheromateux bien que necessaire avant le choix d’une methode contraceptive hormonale, n’est pas systematique sous nos cieux. En Afrique et precisement au Benin, peu de travaux se sont interesses au profil de risque cardiovasculaire des femmes sous contraception hormonale, raison pour laquelle nous avons initie l’etude du risque cardiovasculaire absolu (RCVA) de ces femmes a Porto–Novo.

## Méthode

Nous avons realise une etude descriptive transversale de mars a juin 2016.

Ont ete incluses de facon exhaustive et consecutive toutes les femmes se presentant a l’Association Beninoise pour la Promotion de la Famille (site de Porto–Novo), pour le renouvellement d’une methode hormonale de contraception. La taille minimale predeterminee de l’echantillon, obtenue par la formule de Schwartz etait de 384 sujets.

La collecte des donnees a ete faite par un etudiant en fin de formation en medecine. Une entrevue face a face a eu lieu entre l’enqueteur et l’enquete (en francais ou en langue locale ‘goun’), grace a un questionnaire standardise ecrit en francais. Elle a permis de recueillir les donnees sociodemographiques et comportementales, notamment la recherche d’un tabagisme actif de moins de 12 mois.

Les parametres anthropometriques ont ete mesures (poids, taille, tour de taille). La pression arterielle (PA) a ete prise grace a un tensiometre semi–automatique teste et valide. La glycemie a jeun a ete dosee sur un prelevement veineux sanguin et un electrocardiogramme a ete enregistre en utilisant un appareil C3000 de marque Bionet.

La PA elevee a ete retenue en cas de traitement medicamenteux hypotenseur en cours ou pour une PAS ≥ 140 et/ou une PAD ≥ 90 mmHg. La repartition des chiffres de pression arterielle s’est basee sur la classification de la Societe Europeenne de Cardiologie (ESH/ESC).^3^

L’hyperglycemie de type diabetique a ete retenue en cas de traitement medicamenteux du diabete ou pour une glycemie plasmatique veineuse a jeun ≥ 1.26 g/l.4 L’intolerance au glucose est definie quant a elle par une glycemie a jeun comprise entre 1.02 et 1.25 g/l.

Le tour de taille representant le pourtour abdominal, a ete mesure au niveau d’un point situe a egale distance du bord inferieur de la derniere cote et de la crete iliaque. La mesure etait notee a la fin d’une expiration. Une obesite abdominale a ete retenue pour un tour de taille > 88 cm.^3^

L’obesite et la surcharge ponderale ont ete definies a partir du calcul de l’indice de masse corporelle (IMC) grace a la formule poids (kg)/taille^2^ (m^2^). Les femmes ayant un IMC compris entre 25 et 29.9 kg/m^2^ ont ete considerees comme etant en surcharge ponderale alors que celles ayant un IMC ≥ 30 kg/m^2^ ont ete considerees comme obeses.^3^

L’hypertrophie ventriculaire gauche a ete definie a l’electrocardiogramme (ECG) par un indice de Sokolow–Lyon (SV1 ou SV2 + RV5 ou RV6) ≥ 35 mm ou par un indice de Cornell (SV3 + RVL) ≥ 20 mm.^5^

L’evaluation du risque cardiovasculaire absolu (RCVA) a ete faite, utilisant dans un premier temps sur le diagramme de l’OMS et de la Societe Internationale d’Hypertension Arterielle (WHO/ ISH),^6^ et dans un second temps la methode de l’ESH/ESC.^2^ La methode WHO/ISH a permis de classer les femmes selon leur RCVA en cinq categories (< 10%, entre 10 et 20%, entre 20 et 30%, entre 30 et 40% et > 40%). La methode ESC/ESH a permis quant elle, de les classer en cinq categories de risque (faible, faible a modere, modere, modere a eleve et eleve).^7^ Ces pourcentages definissent la probabilite de presenter au cours des 10 annees suivantes, un accident cardiovasculaire majeur fatal ou non.

La saisie et l’analyse des donnees ont ete faites sur logiciel Epi–Info 3.1. Les variables quantitatives ont ete exprimees en moyenne ± ecart–type. Les comparaisons entre sous–groupes ont ete faites avec un seuil de significativite de 5%.

Les autorisations administratives ont ete obtenues aupres des responsables de l’ABPF Porto–Novo. Le consentement ecrit des femmes a ete obtenu avant inclusion dans l’enquete. Les donnees des femmes ont ete collectees et traitees en toute confidentialite.

## Résultats

L’enquete a porte sur 375 femmes soit 97.7% de la taille minimale predeterminee de l’echantillon. Elles etaient agees en moyenne de 35.3 ± 8.2 ans avec des extremes de 18 et 55 ans.

Les facteurs de risque cardiovasculaire: La consommation de tabac au cours des 12 precedents mois a ete identifiee chez une femme soit une prevalence de 0.3%. L’obesite etait retrouvee dans 23.2% des cas (tableau 1). L’obesite abdominale a ete retrouvee chez 47.5% des enquetees (178 cas).

**Tableau 1:** Répartition des femmes selon l’IMC (kg/m^2^), enquête risque cardiovasculaire absolu (RCVA) femmes sous contraceptifs, Porto-Novo 2016

*l’IMC (kg/m^2^)*	*Effectif*	*%*	*% cumulé*
Maigreur (< 18.5)	5	1.3	1.3
Normal (18.5–24.9)	172	45.9	47.2
Surcharge (25.0–29.9)	111	29.6	76.8
Obèse (≥ 30)	87	23.2	100
Total	375		

Le diabete de type 2 a ete retrouve chez 1.5% (6 cas) des enquetees tandis que l’intolerance au glucose etait presente chez 3.1% (11 cas) d’entre elles.

Le tableau 2 montre la repartition des femmes en fonction de leur pression arterielle. Le pourcentage cumule des 3 grades d’hypertension arterielle donne 24% d’enquetees a PA elevee.

**Tableau 2:** Répartition des femmes en fonction de la pression artérielle selon la classification de l’ESH/ESC, enquête risque cardiovasculaire absolu (RCVA) femmes sous contraceptifs, Porto-Novo 2016

*La pression artérielle*	*Effectif*	*%*	*% cumulé*
Optimale	88	23.5	23.5
Normale	110	29.3	52.8
Normale haute	87	23.2	76
HTA grade 1	49	13.1	89.1
HTA grade 2	30	8.0	97.1
HTA grade 3	11	2.9	100
Total	375		

HTA = hypertension

L’hypertrophie ventriculaire gauche a ete diagnostiquee chez 27 femmes soit 7.1%.

Risque cardiovasculaire absolu: Selon la methode WHO/ISH, 97.6% (319 femmes) des enquetees avaient un risque < 10 et 2.4% (8 femmes) un risque compris entre 10 et 20%.

Le tableau 3 montre la repartition du RCVA des enquetees, selon la methode ESH/ESC.

**Tableau 3:** Répartition du risque cardiovasculaire absolu (RCVA) des enquêtées, selon la méthode ESC/ESH, enquête RCVA femmes sous contraceptifs, Porto-Novo 2016

			*Effectif (%)*			
*RCVA*	*PA normale et optimale*	*PA normale haute*	*HTA grade 1*	*HTA grade 2*	*HTA grade 3*	*Total*
Pas d’autre FRCV	85 (27.3)	25 (8)	17 (5.5)	6 (1.9)	3 (1)	136 (43.7)
1 ou 2 FRCV	68 (21.9)	35 (11.2)	17 (5.5)	15 (4.8)	4 (1.3)	139 (44.7)
≥ 3 FRCV ou hyperglycémie ou HVG	15 (4.8)	6 (1.9)	6 (1.9)	5 (1.6)	4 (1.3)	36 (11.6)
Total	168 (54)	66 (21.2)	40 (12.9)	26 (8.4)	11 (3.5)	311 (100.00)

HTA = hypertension, PA = la pression artérielle, HVG = hypertrophie ventriculaire gauche, FRCV = facteur de risque cardiovasculaire, encadré vert-foncé = risque faible, encadré vert-clair = risque faible à modéré, encadré jaune = risque modéré, encadré orange = risque modéré à élevé, encadré rouge = risque élevé.

L’analyse de ce tableau montre que le RCVA etait faible chez 73.9% des enquetees, faible a modere chez 6.7%, modere chez 7.4% des cas, modere a eleve chez 8.6% et eleve chez 5.2% d’entre elles.

## Discussion

La prevalence des facteurs de risque atheromateux a ete recherchee sur une population feminine jeune non menopausee. Dans cette population, nous avons obtenu 24% de femmes a pression arterielle elevee, 1.5% ayant une hyperglycemie, 26.9% en surcharge ponderale, 23.2% d’obeses et 47.5% ayant une obesite abdominale. L’etude de ces parametres en population generale feminine au Benin a rapporte des chiffres similaires concernant la pression arterielle elevee (26.3%) et l’hyperglycemie (2%) mais moins eleves pour ce qui est de la surcharge ponderale (23%) et de l’obesite (14%).^8^

La determination du RCVA basee sur les facteurs de risque etudies et sur l’hypertrophie ventriculaire gauche, a donne selon la methode de l’ESC, un risque modere a eleve dans 8.6% des cas et un risque eleve dans 5.2% des cas. Il a ete demontre que la prise d’hormones contraceptives induit une modification du metabolisme des hydrates de carbone, elle–meme impliquee dans la prise de poids.^2^

Soule au Nigeria a trouve que la prise du poids en trois ans etait plus importante chez des femmes utilisant une contraception hormonale que chez celles utilisant une methode non hormonale.^9^ Asare au Ghana a demontre au cours d’une etude cas temoins que l’usage de contraceptifs hormonaux entrainait de facon significative une prise de poids, une augmentation des taux sanguins de cholesterol total et low–density lipoprotein (LDL) cholesterol ainsi qu’une elevation de la PA diastolique.^10^

L’interaction entre la contraception hormonale et le risque cardiovasculaire a amene l’Organisation Mondiale de la Sante (OMS) a publier des criteres d’eligibilite a l’usage de cette contraception.^11^ Ces criteres ont fait entre autres, du risque cardiovasculaire eleve et de la maladie thromboembolique veineuse, des situations au cours desquelles le risque encouru sous hormone contraceptive est superieur au benefice attendu. Les femmes a risque cardiovasculaire eleve devraient beneficier d’une prise en charge adequate, et surtout beneficier d’une methode contraceptive respectant les criteres d’eligibilite cardiovasculaire etablis.

Selon la methode individuelle de l’OMS, aucune femme enquetee, n’avait un risque superieur a 20% de developper au cours des 10 annees suivantes un evenement cardiovasculaire majeur. Cette difference de resultat ne devrait pas etre percue comme une reelle discordance au vu des items utilises pour chaque outil d’evaluation du risque.

## Conclusion

Le RCVA de cette population de femmes sous hormones contraceptives est eleve chez 5.2% d’entre elles. Ce resultat pose la question de l’eligibilite cardiovasculaire a l’usage des hormones contraceptives dans cette sous–population. Des travaux de recherche seront envisages afin d’etudier de facon plus precise cette eligibilite.

## References

[1] Kearney PM, Whelton M, Reynolds K, Muntner P, Whelton PK, He J (2005). Global burden of hypertension: analysis of worldwide data. Lancet.

[2] Petersen KR, Skouby SO, Pedersen RG (1991). Desogestrel and gestodene in oral contraceptives: 12 months assessment of carbohydrate and lipoprotein metabolism. Obstet Gynecol.

[3] Junquero D, Rival Y (2005). Syndrome métabolique: quelle définition pour quel(s) traitement(s)?. Médecine/Sciences.

[4] IDF Global Guideline for Managing Older People with Type 2 Diabetes [Internet]. International Diabetes Federation. [cited 2017 Jan 24]. Available from: http://www.idf.org/guidelines-older-people-type-2-diabetes.

[5] Casale PN, Devereux RB, Alonso DR, Campo E, Kligfield P (1987). Improved sex-specific criteria of left ventricular hypertrophy for clinical and computer interpretation of electrocadiograms: validation with autopsy findings. Circulation.

[6] (2013). The Task Force for the management of arterial hypertension of the European Society of Hypertension (ESH) and of the European Society of Cardiology (ESC). 2013 ESH/ESC Guidelines for the management of arterial hypertension. Eur Heart J.

[7] World Health Organization/International Society for Hypertension. Pocket guidelines for Assessment and Management of Cardiovascular Risk with WHO/ISH Cardiovascular Risk Prediction Charts for WHO epidemiological Sub-regions SEAR-D.; 2011. Available from: http:// ish-world.com/downloads/activities/colour_charts_24_Aug_07.pdf> (last accessed: 14-12-16). http://ish-world.com/downloads/activities/colour_charts_24_Aug_07.pdf.

[8] Houinato DS, Gbary AR, Houehanou YC, Djrolo F, Amoussou M, Segnon-Agueh J (2012). Prevalence of hypertension and associated risk factors in Benin. Rev Epidémiol Santé Publique.

[9] Sule S, Shittu O (2005). Weight changes in clients on hormonal contraceptives in Zaria, Nigeria. Afr J Reproduct Health.

[10] Asare GA, Santa S, Ngala RA, Asiedu B, Afriyie D, Amoah AG (2014). Effect of hormonal contraceptives on lipid profile and the risk indices for cardiovascular disease in a Ghanaian community. Int J Womens Health.

[11] WHO | Medical eligibility criteria for contraceptive use [Internet]. [cited 2016 Dec 14]. Available from:http://www.who.int/reproductivehealth/publications/family_planning/Ex-Summ-MEC-5/en/. http://www.who.int/reproductivehealth/publications/family_planning/Ex-Summ-MEC-5/en/.

